# Sensory Tricks in Dystonia: A Systematic Review and Nested Quantitative Synthesis

**DOI:** 10.1002/brb3.71575

**Published:** 2026-07-14

**Authors:** Anish Mehta, Thyagarajan Shivashanmugam, Michiko K. Bruno, Roongroj Bhidayasiri, Sanjay Pandey, Kailash P. Bhatia, Louis C. S. Tan, Pramod Kumar Pal

**Affiliations:** ^1^ Department of Neurology, Ramaiah Medical College and Hospitals Ramaiah University of Applied Sciences Bengaluru India; ^2^ Department of Research Placeboes Research Foundation Bengaluru India; ^3^ Parkinson's and Movement Disorder Center, The Queen's Medical Center University of Hawaiʻi Honolulu, Hawaii USA; ^4^ John A. Burns School of Medicine University of Hawaiʻi Honolulu, Hawaii USA; ^5^ Department of Medicine Faculty of Medicine Chulalongkorn University Bangkok Thailand; ^6^ Department of Neurology and Stroke Medicine Amrita Institute of Medical Sciences, Amrita Vishwa Vidyapeetham Faridabad India; ^7^ Department of Clinical and Movement Neurosciences UCL Queen Square Institute of Neurology London UK; ^8^ Department of Neurology National Neuroscience Institute Singapore Singapore; ^9^ Department of Neurology National Institute of Mental Health and Neurosciences (NIMHANS) Bengaluru India

**Keywords:** dystonia, geste antagoniste, neuromodulation, quantitative synthesis, sensorimotor integration, sensory tricks, systematic review

## Abstract

**Background:**

Sensory tricks (also termed “alleviating maneuvers”) are voluntary maneuvers that transiently alleviate dystonic postures or movements and represent a hallmark clinical feature of dystonia with diagnostic and mechanistic significance. Despite extensive phenomenological description, the evidence base has not been systematically synthesized.

**Objectives:**

To characterize the prevalence, phenomenology, clinical effects, predictors, neurobiological mechanisms, and translational relevance of alleviating maneuvers in dystonia, and to prospectively evaluate the feasibility of quantitative synthesis across predefined outcome domains.

**Methods:**

A systematic review with a prespecified nested quantitative synthesis was conducted using a preregistered PROSPERO protocol (CRD420251175065). The nested component was defined a priori as a domain‐restricted analytic strategy in which random‐effects meta‐analysis was planned where ≥ 3 conceptually comparable studies were available, and in which the feasibility of pooling was itself treated as a primary methodological outcome. PubMed, Scopus, and the Cochrane Library were searched from inception. Studies reporting original empirical data on alleviating maneuvers in human dystonia of any etiology were included. Qualitative synthesis examined phenomenology, clinical effects, predictors, mechanisms, and translational applications. Heterogeneity was examined separately along clinical, methodological, and outcome‐level dimensions.

**Results:**

Of 631 records, 53 studies met inclusion criteria; 31 contributed to qualitative synthesis and 26 to nested quantitative analyses, with 4 contributing to both. No eligible studies in functional or tardive dystonia were identified. Alleviating maneuvers were reported across dystonia subtypes, with prevalence ranging from 13% to 90%; the largest registry cohort (n = 1477) reported effective maneuvers in 68.7% of patients. Acute motor improvements of 30%–50% in head deviation were reported during trick execution, although effects were transient. Responsiveness was associated with shorter disease duration and better response to botulinum toxin. No domain met criteria for formal meta‐analysis; structural barriers were predominantly methodological and outcome‐level rather than clinical.

**Conclusions:**

Alleviating maneuvers are a clinically consistent and mechanistically informative feature of dystonia, but their magnitude, durability, and predictors remain inconsistently measured. Standardized paradigms and outcome reporting are required to enable quantitative synthesis and sham‐controlled evaluation of device‐based alleviating‐maneuver analogues.

## Graphical abstract

Sensory tricks transiently suppress dystonic movements in up to 90% of patients when systematically elicited, yet remain underyrecognized clinically. Their neurophysiological basis and deviceybased analogues offer a promising, underutilized therapeutic avenue warranting standardized assessment and controlled trials.

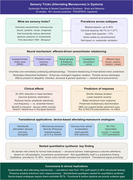

## 1 Introduction

Sensory tricks, or geste antagoniste—increasingly referred to in the contemporary literature as *alleviating maneuvers* to reflect the recognition that these are deliberate sensorimotor behaviors rather than “tricks” in any colloquial sense—are voluntary maneuvers—most commonly light touch, postural adjustments, or other self‐directed sensory actions—that transiently alleviate dystonic postures or movements. First described by Brissaud in 1894 and later formalized by Meige and Feindel in their 1902 monograph on movement disorders, sensory tricks have long been recognized as a hallmark clinical feature of dystonia (Broussolle et al. 2015). For continuity with the longstanding clinical and research literature, we use the terms *sensory trick* and *alleviating maneuver* interchangeably throughout this review, with a preference for the latter where contemporary usage predominates. Their presence has traditionally served as a diagnostic clue distinguishing primary dystonia from psychogenic movement disorders, although this distinction is not absolute.

Sensory tricks occur across focal dystonia subtypes, including cervical dystonia, blepharospasm, oromandibular dystonia, Meige syndrome, laryngeal dystonia, focal hand dystonia, and segmental cranial dystonias (Poisson et al. 2012). While tactile maneuvers involving light touch to the face or head are most common, proprioceptive, visual, cognitive, and imagined sensory strategies have also been described (Counihan and Martino 2020). Sensory tricks are not restricted to idiopathic dystonia and have been reported in secondary and hereditary disorders, including pantothenate kinase–associated neurodegeneration, suggesting a network‐level sensorimotor phenomenon rather than a lesion‐specific mechanism (Martins et al. 2019).

Recognition of sensory tricks has contributed to evolving concepts of dystonia pathophysiology. Dystonia is now widely understood as a disorder of abnormal sensorimotor integration rather than purely aberrant motor output (Prasad et al. 2023). Neurophysiological and neuroimaging studies implicate distributed cortical–subcortical networks—including the basal ganglia, thalamus, supplementary motor area, parietal cortex, and cerebellum—in mediating sensory trick effects (Abbruzzese and Berardelli 2003). Electrophysiological studies further demonstrate modulation of intracortical excitability, motor preparatory activity, and somatosensory gating during sensory trick execution (Amadio et al. 2014). These findings support models in which sensory tricks transiently rebalance mismatched motor predictions and sensory feedback within dysfunctional sensorimotor circuits.

Sensory tricks also have clinical implications. Their presence has been associated with greater responsiveness to botulinum toxin therapy and improved quality‐of‐life measures in patients with dystonia (Mahajan et al. 2023). The transient but reproducible effects of sensory tricks have additionally inspired translational approaches aimed at replicating the underlying afferent inputs, including vibrotactile stimulation (Zhu et al. 2021), mechanical braces (Navrotchi and Badea 2017), sensory‐trick splints (Ramos et al. 2014), and related device‐based neuromodulatory interventions.

Despite more than a century of clinical recognition, the evidence base surrounding sensory tricks remains fragmented. Studies range from case reports and small experimental investigations to registry‐based analyses and employ heterogeneous methods of ascertainment and outcome measurement. Reported prevalence varies widely depending on dystonia phenotype and ascertainment strategy, ranging from approximately 13% with spontaneous self‐report to over 90% when systematic experimental probing is performed (Dagostino et al. 2019; Pandey et al. 2017; Norris et al. 2016). This heterogeneity has limited quantitative synthesis, and to our knowledge no comprehensive systematic review of sensory trick outcomes has been reported.

To address these limitations, we conducted a systematic review with a prespecified nested quantitative synthesis. The objectives were to characterize the prevalence, phenomenology, clinical effects, predictors of response, neurobiological mechanisms, and translational applications of sensory tricks in dystonia and to evaluate the feasibility of quantitative aggregation across the existing literature. In addition to synthesizing existing evidence, this study explicitly examines the quantifiability of the sensory trick literature by testing predefined domains for meta‐analytic feasibility. Where comparable data permitted, quantitative synthesis was performed; where pooling was not feasible, sources of non‐quantifiability were explicitly documented to inform future study design.

## 2 Methods

### 2.1 Study Design

This study was conducted as a systematic review integrating a qualitative synthesis with a prespecified nested quantitative synthesis, according to a preregistered PROSPERO protocol (CRD420251175065). In this review, “nested quantitative synthesis” refers to a prespecified, domain‐restricted analytic component embedded within an overarching qualitative systematic review in which random‐effects meta‐analysis was planned for predefined outcome domains whenever ≥ 3 studies reported conceptually and methodologically comparable outcomes and in which the feasibility of quantitative pooling was itself treated as a primary methodological outcome. This approach extends the SWiM framework by combining narrative synthesis with an a priori, domain‐by‐domain evaluation of quantifiability: rather than treating the absence of pooling as a downstream consequence of heterogeneity, the protocol explicitly tested each predefined domain against prespecified pooling criteria and documented the specific structural barriers (outcome definition, reporting completeness, study design, and ascertainment method) that precluded aggregation. The nested quantitative component therefore serves a dual purpose—generating pooled estimates where feasible and providing a structured, prespecified characterization of the boundaries of quantifiability across the sensory trick literature.

Random‐effects meta‐analysis was prespecified for domains in which three or more studies reported sufficiently comparable outcomes. Given the anticipated clinical and methodological heterogeneity of the sensory trick literature, qualitative synthesis was prioritized as the primary analytic framework. Where substantial heterogeneity in outcome measurement, study design, or reporting structure precluded valid statistical pooling, findings were synthesized narratively in accordance with SWiM (Synthesis Without Meta‐analysis) guidance (Campbell et al. 2020). Publication bias assessment using funnel plots and Egger's test was prespecified for any domain meeting the ≥ 10‐study threshold recommended by Cochrane; because no domain met both the pooling threshold and the minimum study count, formal publication bias testing was not undertaken, and this limitation is acknowledged explicitly.

### 2.2 Search Strategy and Study Selection

A comprehensive literature search was conducted in PubMed, Scopus, and the Cochrane Library from database inception to the final search date using terms related to dystonia, sensory tricks (geste antagoniste), sensory modulation, and neuromodulatory strategies (Table S1). Only English‐language studies were included. Reference lists of included articles and relevant reviews were also screened to identify additional eligible studies. Embase was not included because institutional access was not available to the review team; this decision was made a priori and documented in the PROSPERO protocol. To mitigate the resulting risk of incomplete retrieval, Scopus, which indexes a large fraction of biomedical content overlapping with Embase, including European and device‐related literature, was searched in parallel, and reference lists of all included studies and relevant narrative reviews were hand‐searched.

Study selection was performed in two stages. Titles and abstracts were independently screened by TS and AM, followed by full‐text assessment against predefined eligibility criteria. Disagreements were resolved through discussion and consensus.

### 2.3 Eligibility Criteria

Studies were eligible if they involved human participants with dystonia and reported original empirical data on sensory tricks or sensory trick–like phenomena (also termed alleviating maneuvers; see Results for terminology). Eligible designs included observational clinical studies (cross‐sectional and cohort), registry analyses, neurophysiological or neuroimaging investigations, interventional or device‐based studies, case series, and case reports. No restrictions were placed on dystonia etiology; idiopathic, inherited, acquired, functional, and tardive forms of dystonia were all eligible for inclusion. In practice, the retrieved literature comprised predominantly idiopathic and inherited dystonias, with no eligible studies identified that systematically evaluated sensory tricks in functional or tardive dystonia; this distribution is reported transparently in the Results and revisited in the Discussion as an evidence gap rather than a protocol exclusion.

For the qualitative synthesis, studies were included if they addressed at least one predefined domain: phenomenology and classification of sensory tricks, clinical effects (including acute efficacy or durability), predictors of responsiveness, neurobiological mechanisms, or translational applications.

For the prespecified nested quantitative synthesis, studies were required to evaluate externally applied afferent modulation strategies intended to reproduce sensory trick like effects and to report extractable quantitative motor outcomes. This restriction was applied a priori for two reasons. First, externally applied afferent modulation provides a defined, reproducible stimulus parameter (modality, location, intensity, and duration) that supports cross‐study comparability, whereas spontaneously discovered volitional sensory tricks vary in form, force, and timing across individuals and are rarely operationalized identically between studies. Second, externally applied paradigms allow standardized on–off contrasts and instrumented motor outcomes, which are prerequisites for pooled effect‐size estimation. We acknowledge that this restriction excludes the larger body of observational and phenomenological literature on volitional sensory tricks; that literature was retained for qualitative synthesis, and its non‐quantifiability was treated as a substantive finding rather than a preemptively excluded category. Randomized and non‐randomized interventional studies, as well as observational studies using instrument‐based motor measures, were eligible for quantitative aggregation. Studies lacking quantifiable motor outcomes, examining purely volitional sensory tricks without externally applied modulation, or reporting non‐comparable outcome metrics were excluded from quantitative synthesis but retained for qualitative analysis where relevant.

Narrative reviews, editorials, commentaries, and conference abstracts without primary data were excluded. No restrictions were applied based on dystonia subtype, age, or sex. Only English‐language studies were included.

### 2.4 Data Extraction and Outcome Domains

Data were extracted independently by TS and AM using a structured framework. Extracted variables included study design, dystonia subtype, participant characteristics, sensory trick modality, outcome measures, timing and durability of effects, predictors of response, and relevant mechanistic or translational findings. Discrepancies were resolved through discussion and consensus.

For the qualitative synthesis, findings were analyzed within prespecified outcome domains corresponding to the protocol objectives, with emphasis on convergence across studies and sources of heterogeneity. For the prespecified nested quantitative synthesis, additional data were extracted from studies evaluating externally applied afferent modulation strategies intended to reproduce sensory trick–like effects. Extracted variables included quantitative motor outcomes, sample size, and measures of variance. Quantitative pooling was performed only when outcomes were conceptually and methodologically comparable; otherwise, findings were synthesized narratively.

### 2.5 Risk of Bias Assessment

Risk of bias was assessed for all studies included in the qualitative and quantitative syntheses using design‐appropriate methodological appraisal tools. Observational cohort and cross‐sectional studies were evaluated using the Newcastle–Ottawa Scale (NOS) (Carra et al. 2025). Experimental and interventional studies were assessed using adapted Cochrane risk‐of‐bias criteria (Sterne et al. 2019), focusing on selection, performance, detection, and reporting bias domains. Case series and case reports were appraised using a modified methodological quality checklist appropriate for descriptive clinical evidence.

Two reviewers (A.M. and T.S.) independently assessed methodological quality, and disagreements were resolved through discussion and consensus. Risk‐of‐bias ratings were summarized across domains and study designs. Risk‐of‐bias judgements were carried forward into the interpretive synthesis, with each quantitative domain re‐evaluated for the predominant bias pattern of its contributing studies; domain‐level confidence in findings was downgraded narratively where bias patterns systematically threatened internal validity (e.g., absence of blinding or sham control in device‐based interventional studies, or absence of on–off contrast in observational studies of acute motor effects).

### 2.6 Qualitative Analysis

Qualitative synthesis constituted the primary analytic approach, consistent with the prespecified protocol. A structured narrative framework was used to integrate findings across predefined outcome domains, dystonia phenotypes, sensory trick modalities, and mechanistic frameworks. The synthesis emphasized identification of convergent findings, sources of heterogeneity, and structural factors limiting quantitative aggregation. Patterns observed in the qualitative analysis informed selection of domains for prespecified nested quantitative evaluation.

### 2.7 Quantitative Analysis

Quantitative synthesis was undertaken as a prespecified secondary component and restricted to domains with sufficient conceptual coherence and extractable data. Random‐effects meta‐analysis was planned for domains with ≥ 3 comparable studies. Where pooling was not feasible, reasons for non‐quantifiability—including heterogeneous outcome definitions, inconsistent reporting, and incompatible study designs—were systematically documented, and findings were synthesized in accordance with SWiM guidance (Campbell et al. 2020).

Heterogeneity was examined structurally rather than treated as a single composite barrier to pooling. We prespecified separate consideration of (i) *clinical heterogeneity* (variation in dystonia phenotype, anatomical distribution, disease duration, and etiology); (ii) *methodological heterogeneity* (study design, randomization, blinding, ascertainment strategy, and operational definition of a sensory trick); and (iii) *outcome‐level heterogeneity* (binary vs. continuous outcomes, on–off contrast structure, instrumented vs. clinician‐rated measures, and follow‐up duration). For each predefined quantitative domain, we evaluated whether subgroup‐restricted pooling (e.g., limited to cervical dystonia, or limited to vibrotactile interventions with instrumented kinematic outcomes) would yield a methodologically defensible subset of ≥ 3 studies. Where no such subset existed, the structural reason was documented domain‐by‐domain in the Results, and findings were carried forward into narrative synthesis stratified by the heterogeneity dimensions above. This structured approach is reflected in the organization of Table 2 and in the domain‐specific reporting.

## 3 Results

### 3.1 Study Selection

**FIGURE 1:**
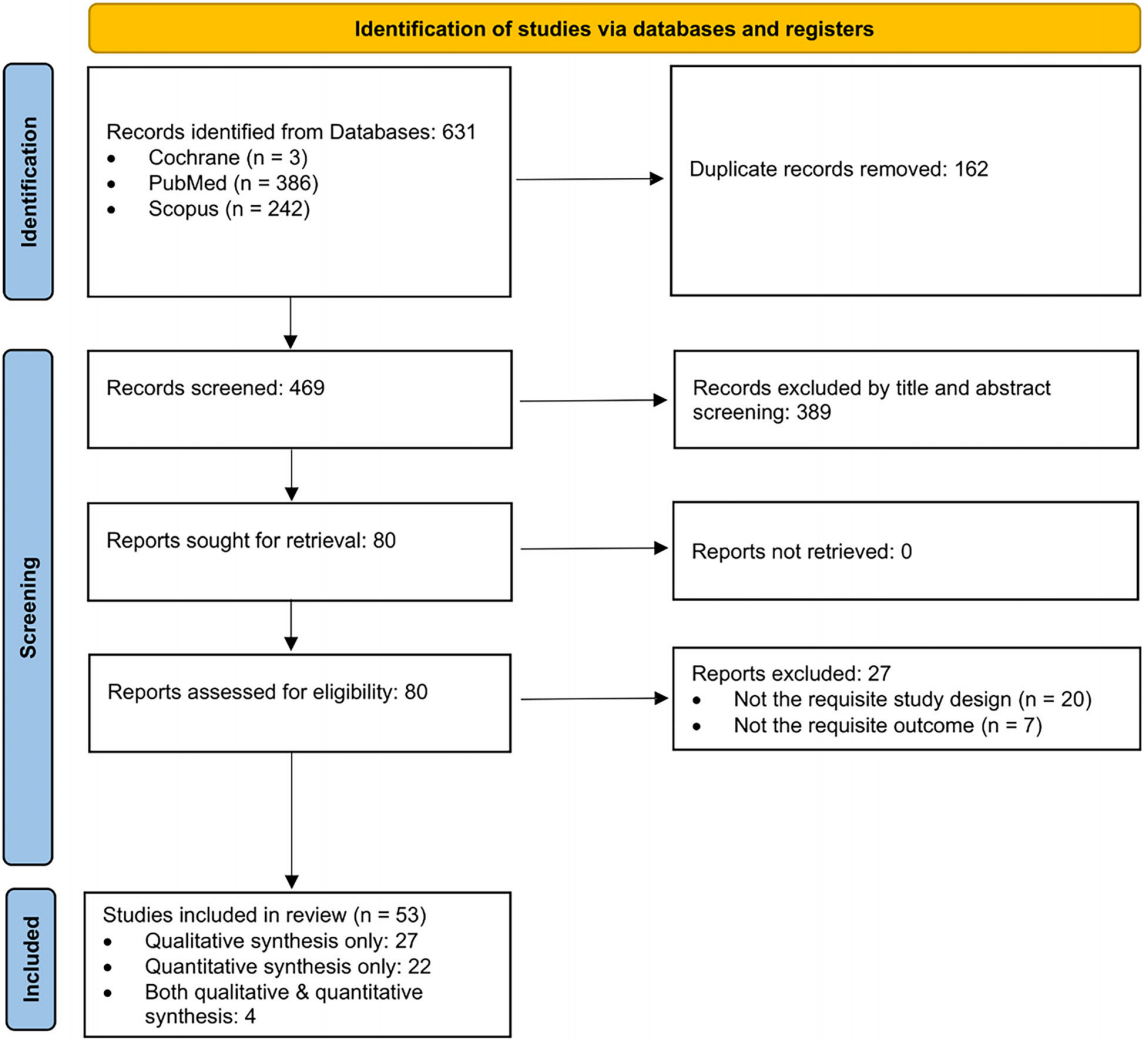
PRISMA flow diagram illustrating the study selection process for the systematic review.

The database search identified 631 records from PubMed (*n* = 386), Scopus (*n* = 242), and the Cochrane Library (*n* = 3). After removal of 162 duplicates, 469 records remained. Title and abstract screening excluded 389 records, leaving 80 articles for full‐text assessment. Of these, 27 were excluded, resulting in 53 studies meeting the eligibility criteria. Of the included studies, 31 contributed to qualitative synthesis and 26 to nested quantitative analyses, with four studies contributing to both. The study selection process is illustrated in the PRISMA (Page et al. 2021) flow diagram (Figure 1).

### 3.2 Characteristics of Included Studies

The included studies comprised a heterogeneous body of evidence spanning multiple designs and dystonia phenotypes (Table S2). Across studies reporting sample size, the literature encompassed approximately 4000–4500 patients, with individual study sizes ranging from single case reports to large registry‐based cohorts exceeding 1500 participants. Several large registry and cohort studies (*n* > 200) were complemented by multiple intermediate‐sized studies (*n* ≈ 50–200) and smaller experimental or case‐based investigations. Study designs included observational clinical cohorts, registry analyses, neurophysiological and neuroimaging investigations, interventional feasibility or device‐based studies, and case series or case reports. Within this body of evidence, the qualitative synthesis (31 studies) captured the multidimensional phenomenology of sensory tricks, whereas the quantitative component consisted primarily of interventional and device‐based studies evaluating externally applied afferent modulation with extractable motor outcomes.

Cervical dystonia was the most frequently studied phenotype, followed by blepharospasm, oromandibular dystonia, Meige syndrome, focal hand dystonia, laryngeal dystonia, segmental dystonia, and mixed cranial dystonias. Some studies also included secondary or genetic forms of dystonia.

Across studies, sensory tricks were evaluated using clinical examination, patient‐reported assessments, kinematic analysis, electromyography, and neurophysiological techniques. Outcome measures ranged from binary classification of sensory trick presence to quantitative assessments of posture, muscle activity, tremor amplitude, and related physiological parameters.

From this evidence base, 26 studies met the eligibility criteria for the nested quantitative synthesis (Table S2), predominantly evaluating afferent‐based neuromodulation strategies designed to reproduce sensory trick–like effects.

### 3.3 Risk of Bias Assessment

Overall methodological quality across the included studies was moderate, reflecting the predominance of observational and exploratory designs in the sensory trick literature. Observational studies generally demonstrated adequate clinical characterization but often lacked clearly defined control groups or prospective sampling strategies. Neurophysiological and neuroimaging studies showed low measurement bias owing to objective outcome measures such as electromyography, kinematic recordings, and neuroimaging metrics, although many had small sample sizes.

Interventional feasibility and device‐based studies frequently lacked randomization, blinding, or sham control conditions, increasing the risk of performance and detection bias. Case series and case reports were included to capture phenomenological diversity but were interpreted descriptively owing to inherent risks of selection and reporting bias. Detailed study‐level assessments are provided in Table S3.

Across the 53 included studies, overall risk of bias was rated low in 1 study (a single randomized controlled trial of vibrotactile stimulation in laryngeal dystonia (Konczak et al. 2024); moderate in approximately 45 studies (predominantly cross‐sectional, cohort, registry, and small experimental designs); and high in seven case reports or small case series. Selection bias was the most prevalent domain‐level concern, reflecting reliance on convenience sampling and the absence of consecutive recruitment in most cross‐sectional and registry studies. Detection bias was generally moderate because outcome assessors were rarely blinded to sensory trick status, although neurophysiological studies using objective instrumentation (TMS, EMG, kinematic recordings, and neuroimaging) partially mitigated this. Performance bias was concentrated in the interventional and device‐based studies, where sham‐controlled designs were largely absent. Reporting bias was rated low across most studies, although prespecified analysis plans were rare outside the single RCT.

Risk‐of‐bias judgements were carried forward into the domain‐level interpretation of findings as follows. For the *prevalence* domain, moderate selection bias and inconsistent ascertainment methods materially affected confidence, and we therefore present prevalence estimates as context‐dependent rather than as estimates of a single population parameter. For the *acute motor improvement* domain, the absence of standardized on–off paradigms and blinded outcome assessment in the contributing studies reduces confidence in the magnitude of reported effects; the consistency of directionality across studies—but not the precision of effect sizes—should be regarded as the interpretable signal. For the *tactile cutaneous afferent modulation* domain, contributing studies were predominantly moderate‐risk observational designs; the convergent finding of reproducible but partial benefit is therefore interpreted as a robust phenomenological observation rather than as a calibrated effect‐size estimate. For the *device‐based sensory trick analogues* domain, the absence of blinded parallel‐group randomized trials (with the single exception of Konczak et al. 2024) and the predominance of single‐arm or crossover designs without sham control mean that reported responder proportions of 50%–70% should be regarded as upper‐bound estimates that have not been corrected for placebo or expectation effects. For the *mechanistic* synthesis, contributing neurophysiological and neuroimaging studies had low measurement bias but small sample sizes; mechanistic inferences are therefore presented as convergent across studies rather than as confirmed within any single adequately powered investigation. For the *predictors* domain, all contributing studies analyzed binary presence/absence rather than quantified response, and confidence in the magnitude of predictor effects is correspondingly low even where direction of association is consistent.

The aggregate effect of these bias patterns is that the directional findings of the review—that alleviating maneuvers are common across dystonia subtypes, produce acute and transient motor benefit, modulate distributed sensorimotor networks, and have plausible translational potential—are supported with moderate confidence, whereas the *quantitative* estimates of prevalence, effect magnitude, and durability should be regarded as provisional and context‐dependent. This calibration of confidence informs the interpretation in the Discussion and the methodological recommendations that follow.

### 3.4 Qualitative Synthesis/Systematic Review

#### 3.4.1 Phenomenology and Clinical Characteristics

Data from the included studies indicate that sensory tricks (*alleviating maneuvers*; *geste antagoniste*) are a characteristic clinical feature of dystonia (Table 1), consisting of subtle voluntary sensory or motor actions that transiently alleviate abnormal postures or movements (Frucht 2014). Sensory tricks (hereafter used interchangeably with *alleviating maneuvers*) are most frequently reported in cervical dystonia but also occur in blepharospasm, oromandibular dystonia, Meige syndrome, laryngeal dystonia, focal hand dystonia, segmental dystonia, and mixed cranial dystonias (Pandey et al. 2017; Masuhr et al. 2000; Singer and Papapetropoulos 2006; Martino et al. 2010; Pandey and Sharma 2018; Norby et al. 2015).

**TABLE 1:** Emergent themes from qualitative synthesis of sensory tricks in dystonia.

Qualitative theme	Core observations across studies	Contextual modifiers identified	Interpretive insight
**Phenomenological diversity**	Sensory tricks encompass tactile, proprioceptive, visual, cognitive, imagined, and internally generated actions, with marked inter‐individual variability (Martins et al. 2019, Abbruzzese and Berardelli 2003, Pandey et al. 2017, Norris et al. 2016, Avanzino et al. 2025).	Dystonia phenotype, task context, sensory modality, individual experimentation.	Sensory tricks represent a family of compensatory behaviors rather than a unitary phenomenon.
**Voluntary and often subtle nature**	Sensory tricks are voluntary actions that are typically subtle sensory or motor behaviors discovered through patient experimentation (Martins et al. 2019, Abbruzzese and Berardelli 2003, Avanzino et al. 2025).	Learning history, patient awareness, disease duration.	Effectiveness appears to depend on preserved sensorimotor integration rather than purely mechanical effects.
**Transient efficacy**	Clinical benefit is consistently described as immediate and short‐lived, resolving after cessation of the trick (Pandey et al. 2017, Frucht 2014).	Duration of application, continuous vs. intermittent use.	Supports a transient overriding influence on abnormal motor output rather than permanent normalization.
**Phenotype dependence**	Sensory tricks are most prevalent and effective in cervical dystonia but are also described in cranial, laryngeal, and limb dystonias (Martins et al. 2019, Abbruzzese and Berardelli 2003, Avanzino et al. 2025, Kägi et al. 2013).	Distribution of dystonia, focal vs. segmental involvement.	Expression reflects differences in network organization rather than a fixed anatomical lesion location.
**Spontaneous discovery vs. elicitation**	Many patients identify sensory tricks independently, while others require structured probing during clinical examination (Abbruzzese and Berardelli 2003, Avanzino et al. 2025).	Clinical examination style, patient insight, prior exposure to maneuvers.	Ascertainment strategy strongly influences reported prevalence and the apparent absence of sensory tricks.
**Network‐level mechanism**	Qualitative and mechanistic studies converge on distributed cortical–subcortical involvement rather than simple peripheral muscle inhibition (Broussolle et al. 2015, Lee et al. 2012, Yamada et al. 2007, Müller et al. 2001, Schramm et al. 2004, Petrović et al. 2014).	Sensory modality engaged, task relevance.	Sensory tricks likely modulate abnormal motor output through afferent‐driven network modulation.
**Persistence of abnormal physiology**	Abnormal reflexes or neurophysiological markers may persist despite observable clinical improvement during trick execution (Lee et al. 2012, Müller et al. 2001, Petrović et al. 2014).	Type of physiological measure used, experimental paradigm.	Indicates that sensory tricks may compensate for, rather than correct, underlying pathophysiology.
**Context dependence of expression**	Effectiveness of sensory tricks varies across individuals and clinical contexts and may depend on phenotype and disease characteristics (Sterne et al. 2019, Masuhr et al. 2000, Singer and Papapetropoulos 2006).	Dystonia phenotype, disease duration, severity.	Suggests that sensory trick efficacy is influenced by disease state and sensorimotor network configuration.
**Attenuation with disease progression**	Some studies report reduced effectiveness of sensory tricks with increasing disease duration (Frucht 2014, Masuhr et al. 2000).	Disease duration and progression of dystonia severity.	Suggests gradual attenuation of compensatory mechanisms as disease evolves.
**Translational relevance**	Observations of sensory trick phenomena have inspired device‐based approaches and afferent neuromodulation strategies (Zhou et al. 2016, Cai et al. 2024, Matteo et al. 2021, Kilduff et al. 2016, Ehrlich and Frucht 2016, Svetel et al. 2013, Idrissi et al. 2025).	Device design, stimulation parameters, patient phenotype.	Translation is biologically plausible but constrained by heterogeneity of mechanisms and durability of effect.

Registry and cohort studies suggest that sensory tricks are reported in both task‐specific and non–task‐specific dystonia, although their expression varies. Tactile maneuvers involving light touch to the face or head predominate, but proprioceptive, visual, cognitive, imagined, and internally generated sensory strategies have also been described (Greene and Bressman 1998; Boyd et al. 2013; Lee et al. 2012). Sensory tricks are not limited to primary dystonia and have been reported in secondary and hereditary conditions, including pantothenate kinase–associated neurodegeneration and structural thalamic lesions, supporting a network‐level rather than lesion‐specific phenomenon (Yamada et al. 2007).

#### 3.4.2 Clinical Efficacy

Across observational and experimental studies, sensory tricks are associated with consistent but heterogeneous acute motor effects. Quantitative kinematic and electromyographic studies report that, within studied cohorts, a substantial proportion of patients with cervical dystonia experience clinically meaningful reductions in abnormal head deviation during sensory trick execution, frequently exceeding 30%–50% improvement thresholds (Müller et al. 2001; Schramm et al. 2004). In dystonic tremor, sensory tricks selectively reduce tremor amplitude without altering tremor frequency, an effect not observed in essential tremor, supporting both acute symptomatic modulation and phenomenological differentiation (Masuhr et al. 2000).

In blepharospasm, tactile sensory tricks are common and often associated with partial symptom improvement (Pandey et al. 2017). Neurophysiological studies corroborate these clinical observations, demonstrating modulation of abnormal intracortical facilitation and motor cortex excitability during sensory trick application (Amadio et al. 2014). However, efficacy is strongly phenotype‐dependent; in laryngeal dystonia, systematic testing of tactile, vibrotactile, and auditory manipulations failed to improve symptoms and, in some cases, worsened voice quality (Dwenger et al. 2025).

#### 3.4.3 Durability of Benefits

A consistent finding across the literature is that the benefit of sensory tricks is transient. Improvements typically occur immediately during sensory trick execution but decay within minutes following cessation (Müller et al. 2001; Avanzino et al. 2025). Experimental vibrotactile stimulation studies report partial retention of benefit at one to five minutes, with near‐complete loss of effect by approximately twenty minutes (Avanzino et al. 2025). Increasing disease duration is consistently associated with reduced sensory trick effectiveness, suggesting progressive attenuation of compensatory mechanisms (Benadof et al. 2019; Kägi et al. 2013). No included study reports sustained long‐term benefit.

#### 3.4.4 Predictors and Modifiers of Response

Several convergent factors associated with sensory trick responsiveness emerge across studies. Shorter disease duration, lower baseline severity, and preserved multisensory discrimination are associated with more complete or reliable benefit (Kägi et al. 2013; Filip et al. 2016). The presence of a sensory trick is associated with greater responsiveness to botulinum toxin therapy and lower cumulative dose requirements across focal dystonias (Mahajan et al. 2023; Filip et al. 2016; Tomić et al. 2015). Responsiveness appears modifiable by circuit‐level interventions; following pallidal deep brain stimulation, expansion of the tactile catchment area of sensory tricks has been observed.

Genetic association studies do not support a single‐gene explanation. No association was identified between *NALCN* polymorphisms and the presence of sensory tricks in isolated cervical dystonia (Zhou et al. 2016). Sex likewise does not significantly influence the prevalence or effectiveness of sensory tricks (Kilic‐Berkmen et al. 2024).

#### 3.4.5 Mechanistic Insights

Mechanistic investigations consistently support the interpretation that sensory tricks involve active afferent‐driven modulation of abnormal sensorimotor integration, rather than being fully explained by distraction or peripheral muscle inhibition. Studies of sensory gating in focal hand dystonia report abnormal premovement suppression of somatosensory evoked potentials, supporting altered afferent processing (Murase 2000, Lourenço et al. 2007).

**FIGURE 2:**
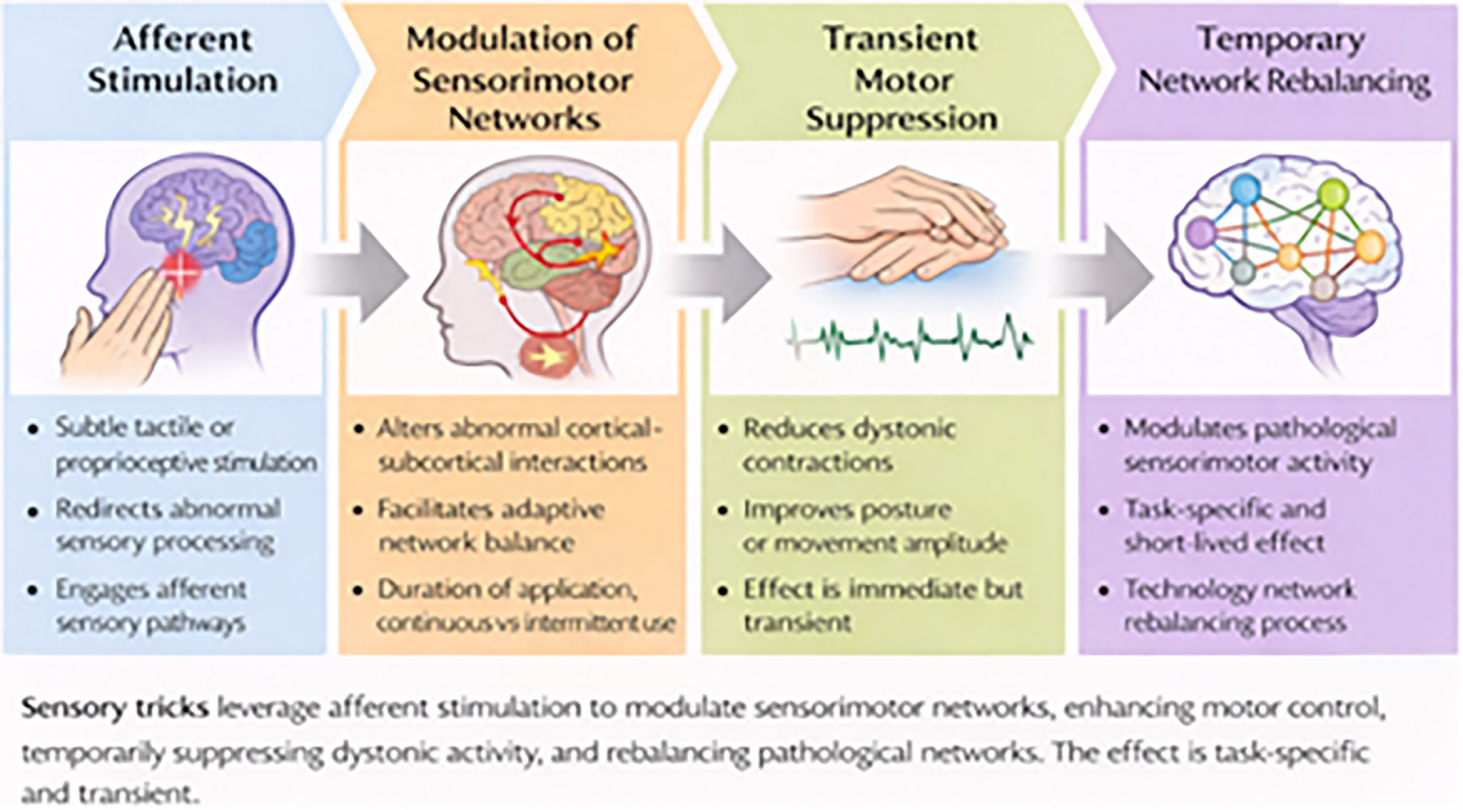
Conceptual framework of sensory trick modulation in dystonia derived from qualitative and mechanistic evidence. Schematic representation summarizing the conceptual model emerging from the literature reviewed in this study. Sensory tricks involve subtle afferent sensory input, such as tactile or proprioceptive stimulation, which can transiently modulate abnormal sensorimotor processing within distributed cortical–subcortical networks. This modulation may temporarily suppress dystonic contractions and improve posture or movement amplitude. The effect is typically task‐specific and short‐lived, reflecting transient rebalancing of dysfunctional sensorimotor network activity rather than permanent correction of the underlying pathophysiology.

Neurophysiological and imaging studies implicate distributed cortical–subcortical networks involving the supplementary motor area, parietal cortex, cerebellum, basal ganglia, and thalamo‐cortical loops (Cho et al. 2022; Cai et al. 2024). Electroencephalographic evidence indicates modulation of motor preparatory activity during sensory trick execution, including enhancement of late contingent negative variation (Shin et al. 2021). Vestibular and brainstem reflex studies show persistence of abnormal responses despite clinical improvement, indicating that sensory tricks override rather than normalize underlying pathophysiology (Mazzini and Schieppati 1994). Collectively, these findings support conceptual models in which sensory tricks temporarily rebalance mismatched motor predictions and sensory feedback within distributed sensorimotor networks (Figure 2) (Frucht 2014).

#### 3.4.6 Measurement Considerations

Assessment of sensory tricks presents methodological challenges. Clinimetric analyses of dystonia rating scales identify insufficient testing duration, rater misinterpretation, and baseline severity effects as sources of measurement error, which may lead to underestimation of sensory trick effects if not accounted for (Cisneros et al. 2020). The recent shift in nomenclature toward *alleviating maneuver*—endorsed in the most recent dystonia rating‐scale clinimetric work (Cisneros et al., 2020)—also has practical implications for assessment, as it discourages dismissive framing and supports systematic elicitation during examination. These findings highlight the importance of standardized assessment procedures in both clinical and research settings.

#### 3.4.7 Themes Eligible for Nested Quantitative Synthesis

Six thematic domains were identified as eligible for nested quantitative synthesis based on recurrence across studies, conceptual coherence, and availability of extractable quantitative outcomes. These included prevalence of sensory tricks, acute motor improvement during trick execution, tactile sensory modulation, proprioceptive or postural sensory tricks, device‐based sensory trick analogues, and short‐term retention of effect. Domains lacking comparable outcome definitions or extractable data were synthesized narratively.

### 3.5 Nested Quantitative Synthesis

Across the evidence base, quantitative pooling was frequently not feasible due to heterogeneity in study design, outcome definitions, and reporting practices. Consistent with the structured approach prespecified in Methods, heterogeneity was examined along three dimensions for each domain: clinical heterogeneity (dystonia phenotype, anatomical distribution, disease duration), methodological heterogeneity (design, ascertainment, randomization, blinding, and operational definition of a sensory trick), and outcome‐level heterogeneity (binary vs. continuous outcomes, on–off contrast structure, instrumented vs. clinician‐rated measurement, and follow‐up duration). For each predefined domain, we evaluated whether a subgroup‐restricted subset of ≥ 3 comparable studies could be assembled for pooling. Where this was not feasible, the specific dimensions of heterogeneity that prevented aggregation are reported below and summarized in Table 3. Reasons for non‐quantifiability—including insufficient reporting of numerators and denominators, incompatible outcome metrics, short or inconsistent assessment windows, and excessive clinical or methodological heterogeneity—were documented and synthesized.

#### 3.5.1 Quantifiability of Sensory Trick Prevalence

The predefined prevalence subset comprised 16 studies assessing sensory tricks in dystonia. In most studies, sensory tricks were not analyzed as a primary outcome but were reported descriptively or as secondary variables within broader clinical or phenotypic analyses. Consequently, only a subset reported absolute numerator–denominator data permitting extraction of prevalence estimates.

Marked variability in reported prevalence was observed across dystonia phenotypes and methods of ascertainment. In one cross‐sectional study, sensory tricks were reported in 90% of patients with primary blepharospasm compared with 35% of those with idiopathic cervical dystonia (Pandey et al. 2017). In contrast, in idiopathic adult‐onset upper limb dystonia, spontaneous sensory tricks were reported in only 13% of cases despite greater responsiveness when maneuvers were experimentally elicited (Dagostino et al. 2019). These findings indicate that prevalence estimates are highly sensitive to both dystonia phenotype and ascertainment method.

The most internally consistent prevalence data derive from the Dystonia Coalition multicentre registry (*n* = 1477 cervical dystonia), in which effective sensory tricks were documented in 68.7% of patients (Norris et al. 2016). Prevalence varied according to onset pattern and anatomical distribution, decreasing in patients with non‐neck onset and subsequent spread.

**FIGURE 3:**
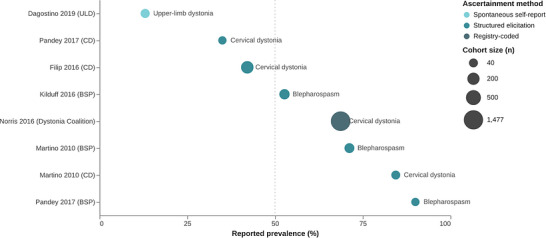
Prevalence of alleviating maneuvers (sensory tricks) in dystonia, by phenotype and ascertainment method. Each point represents one study cohort; point size is proportional to sample size, and color denotes the ascertainment method. The reported prevalence range of 13%–90% is largely attributable to methodological variation in ascertainment and to differences in how an “effective” or “typical” sensory trick is operationalized, rather than to true biological differences between phenotypes. Spontaneous self‐report systematically yields the lowest estimates (Dagostino 2019); structured elicitation produces intermediate‐to‐high values that vary further with the operational definition applied (e.g., Filip 2016 reports 42.1% for the typical light‐touch maneuver and 77% for any postural correction); registry‐coded ascertainment in the largest cohort (Norris 2016, Dystonia Coalition, *n* = 1477) sits centrally. BSP, blepharospasm; CD, cervical dystonia; ULD, upper‐limb dystonia.

Several registry and cohort studies reported sensory tricks as associated clinical features without providing numerator–denominator data (Zhou et al., 2016; Matteo et al. 2021; Kilduff et al. 2016; Ehrlich and Frucht 2016; Zhou et al. 2016; Svetel et al. 2013; Idrissi et al. 2025; Van der et al. 2015). Subgroup‐restricted pooling was evaluated for cervical dystonia specifically (the most consistently reported phenotype). Even within this restricted subset, methodological heterogeneity in ascertainment strategy (spontaneous self‐report vs. structured elicitation vs. registry coding) and outcome‐level heterogeneity in the operational definition of an “effective” sensory trick prevented assembly of a defensible pooling subset of ≥ 3 methodologically comparable studies. Prevalence estimates (Figure 3) are therefore reported as a range stratified by phenotype and ascertainment method (Table 3) rather than as a pooled summary statistic.

#### 3.5.2 Acute Motor Improvement During Sensory Trick Execution

Within the predefined domain examining acute motor effects, six studies were identified that evaluated sensory tricks in dystonia in relation to motor outcomes (Pandey et al. 2017; Cutsforth‐Gregory et al. 2016; Fantato et al. 2019; Lorenzano et al. 2019; Correa‐Vela et al. 2023; Velucci et al. 2025). Following full‐text assessment, none met the predefined eligibility criteria for inclusion in quantitative synthesis of acute motor improvement during sensory trick execution.

Across the identified studies, sensory tricks were evaluated either descriptively, through retrospective self‐report, or within the context of longer‐term functional, therapeutic, or device‐based interventions (Pandey et al. 2017; Fantato et al. 2019; Lorenzano et al. 2019). Where experimental or clinical observational paradigms were employed, outcome reporting was heterogeneous and generally did not permit extraction of comparable same‐session estimates of immediate motor change attributable specifically to sensory trick application (Cutsforth‐Gregory et al. 2016; Correa‐Vela et al. 2023). The structural barriers were concentrated at the methodological and outcome levels: studies variably lacked controlled on–off paradigms, standardized motor outcome measures, or extractable summary statistics, and several embedded sensory trick assessments within longer‐term functional, therapeutic, or device‐based interventions rather than as a primary, isolable contrast (Pandey et al. 2017; Cutsforth‐Gregory et al. 2016; Fantato et al. 2019; Lorenzano et al. 2019; Correa‐Vela et al. 2023; Velucci et al. 2025). Clinical heterogeneity was less limiting in this domain than for prevalence, as five of the six studies focused on cervical or cervical‐predominant cohorts; the binding constraint was therefore methodological rather than clinical (Table 3). Future investigations employing controlled on–off paradigms with standardized motor outcome measures and clearly defined same‐session contrasts will be necessary to enable robust quantitative estimation of acute motor effects.

#### 3.5.3 Cutaneous Afferent Modulation Through Tactile Sensory Tricks

A nested quantitative synthesis was conducted for tactile sensory tricks involving cutaneous afferent modulation, including light touch, externally applied focal pressure, and device‐mediated continuous tactile stimulation. Extractable quantitative data on reproducibility and/or persistence of benefit were available from a small subset of studies, most notably in blepharospasm, cervical dystonia, upper limb dystonia, and selected craniofacial or genetic dystonia cohorts (Dagostino et al. 2019; Pandey et al. 2017; Fantato et al. 2019; Correa‐Vela et al. 2023; Erbguth and Lange 2021). Across these studies, tactile sensory tricks were associated with reproducible but usually partial symptomatic improvement in a subset of patients, although the proportion of responders varied according to dystonia phenotype, stimulation modality, and outcome definition. In a cross‐sectional study of primary blepharospasm and idiopathic cervical dystonia, sensory tricks were reported to be effective on every use in 72.2% of patients with blepharospasm and 85.8% of those with cervical dystonia, although the benefit was partial in most cases (Pandey et al. 2017). In upper limb dystonia, a standardized tactile maneuver consisting of gently grasping the affected wrist improved handwriting in 38% of patients, supporting the reproducibility of a non‐spontaneous tactile trick under structured testing conditions (Dagostino et al. 2019). Available evidence further suggests that benefit generally persists for the duration of the maneuver or task rather than producing sustained carry‐over after stimulation ceases. In children with SGCE‐myoclonus‐dystonia, sensory tricks were reported during writing and walking, indicating task‐linked persistence during action execution, although durability was not formally quantified (Correa‐Vela et al. 2023). In blepharospasm, device‐mediated continuous focal pressure using the Pressop produced some benefit in more than half of trial participants, and 18% experienced substantial improvement, with benefit in some cases reported over follow‐up of up to 5 years (Fantato et al. 2019). By contrast, reports of improvement during face‐mask wearing suggest that naturalistic continuous cutaneous stimulation may act as a sensory trick in a minority of patients with craniofacial dystonia, although these observations were based on self‐reported symptom change rather than formal durability assessment (Erbguth and Lange 2021). Overall, the quantitative evidence supports the view that tactile sensory tricks can produce repeatable, phenotype‐dependent symptomatic benefit, but substantial heterogeneity in study design, stimulation modality, and outcome assessment precluded formal pooling of effect sizes. Accordingly, quantitative findings are best presented descriptively, with studies distinguished according to whether they evaluate repeat‐use reproducibility, task‐bound persistence, or longer‐term benefit during continuous tactile stimulation. Subgroup‐restricted pooling was evaluated separately for three sub‐constructs that the literature conflates—repeat‐use reproducibility, task‐bound persistence during continuous engagement, and longer‐term durability following repeated stimulation. Each sub‐construct was represented by ≤ 2 studies with comparable outcome metrics, falling below the prespecified pooling threshold (Table 3). The principal barrier was outcome‐level heterogeneity: the three sub‐constructs are rarely measured together within a single study, and where measured, use incompatible response definitions. The directional finding—that tactile alleviating maneuvers produce reproducible, phenotype‐dependent, partial benefit confined to the duration of the maneuver or task—is therefore supported across studies but not quantifiable as a single pooled effect estimate.

#### 3.5.4 Proprioceptive and Postural Sensory Tricks

Predefined searches for this domain did not identify any studies that met the eligibility criteria for quantitative synthesis examining predictors of proprioceptive or postural sensory trick effectiveness. Although several observational, cross‐sectional, and registry‐based studies reported associations between sensory tricks and demographic, clinical, phenotypic, or systemic variables, these analyses were based on the binary presence or absence of sensory tricks rather than objectively measured motor responses during execution. Predictors were therefore evaluated in the available literature in relation to whether a sensory trick was reported by patients, rather than in relation to quantifiable motor improvement, task‐specific performance change, or durability of benefit over time (Table 3). Consequently, the available literature does not currently permit estimation of clinical, phenotypic, or biological predictors of sensory trick responsiveness or durability in this domain. Future studies using standardized experimental paradigms, objective motor outcome measures, and longitudinal or repeated‐measures designs will be necessary to determine predictors of proprioceptive or postural sensory trick effectiveness.

#### 3.5.5 Device‐Based Sensory Trick Analoges

A total of 11 interventional studies were included in this exploratory nested synthesis evaluating device‐based sensory trick analogues and externally applied peripheral sensory modulation in dystonia (Amadio et al. 2014; Zhu et al. 2021; Avanzino et al. 2025; Fantato et al. 2019; Lorenzano et al. 2019; Frucht et al. 1999; Yoshida 2018; Nishida et al. 2023; Xu et al. 2024; Konczak et al. 2024; Kasiri et al. 2025). Collectively, these studies enrolled approximately 260–280 participants, predominantly with focal dystonia, including laryngeal (spasmodic dysphonia), cervical, oromandibular, and task‐specific limb dystonia (Fantato et al. 2019; Frucht et al. 1999; Yoshida 2018; Konczak et al. 2024).

The evidence base consisted primarily of early‐phase interventional designs, including prospective single‐group studies and crossover trials; no blinded parallel‐group randomized controlled trials were identified (Fantato et al. 2019; Lorenzano et al. 2019; Frucht et al. 1999; Yoshida 2018; Xu et al. 2024). Interventions included vibrotactile stimulation devices, mechanical or wearable sensory‐trick analogs, and sensorymotor retuning approaches employing splints or task‐specific modulation (Zhu et al. 2021; Frucht et al. 1999; Yoshida 2018; Konczak et al. 2024; Kasiri et al. 2025).

Across studies, 7 of 11 reported clinically or statistically meaningful short‐term improvements in dystonia severity, task performance, or objective physiological measures (Zhu et al. 2021; Fantato et al. 2019; Lorenzano et al. 2019; Frucht et al. 1999; Yoshida 2018; Xu et al. 2024; Konczak et al. 2024). Where responder analyses were reported, response proportions ranged from approximately 50% to 70%, although response definitions varied substantially across studies (Fantato et al. 2019; Yoshida 2018; Konczak et al. 2024). Observed benefits were predominantly transient, typically persisting from minutes to hours, with limited evidence for persistence beyond 24 h and no consistent demonstration of sustained long‐term effects (Fantato et al. 2019; Frucht et al. 1999; Xu et al. 2024). Device‐based sensory modulation was generally well tolerated, with no serious adverse events reported and only minor, self‐limited discomfort observed in a small number of participants (Fantato et al. 2019; Xu et al. 2024). Owing to substantial heterogeneity in intervention characteristics, dystonia phenotypes, outcome measures, and follow‐up durations, formal quantitative pooling of effect sizes was not feasible. Accordingly, no summary estimate of effect magnitude or statistical heterogeneity is reported. Subgroup‐restricted pooling was evaluated for vibrotactile interventions specifically (the largest single‐modality cluster), but the three contributing studies (Zhu et al., 2021; Avanzino et al., 2025; Xu et al., 2024; Konczak et al., 2024) targeted different anatomical sites (neck vs. larynx), used different stimulation parameters, and reported different primary outcomes (motor severity vs. voice quality vs. pain), so even within a single modality the methodological and outcome‐level heterogeneity precluded valid pooling (Table 3). The directional finding—that device‐based alleviating maneuvers produce short‐term benefit in a substantial minority to majority of patients with acceptable tolerability—is supported across the evidence base, but the absence of blinded sham‐controlled designs in all but one study (Konczak et al., 2024) means that the reported 50%–70% responder proportions should be regarded as upper‐bound estimates pending randomized confirmation.

#### 3.5.6 Short‐Term Retention of Effect

Predefined searches for this domain did not identify any studies that systematically evaluated short‐term retention of sensory trick effects or safety outcomes using standardized or predefined outcome measures. Although sensory tricks and device‐mediated tactile stimulation were frequently described as safe or well tolerated in clinical reports and interventional studies, these observations were typically incidental to primary efficacy outcomes and were not assessed using structured safety monitoring frameworks. Across the available literature, no study prospectively evaluated adverse events, unintended effects, or risks associated with repeated or prolonged sensory trick use. Similarly, no investigations assessed short‐term retention of benefit following repeated sensory trick application using standardized experimental paradigms or quantitative outcome measures. Consequently, no studies met eligibility criteria for quantitative synthesis; current evidence therefore does not permit estimation of safety profiles or short‐term durability of benefit associated with sensory trick use. The principal barrier in this domain is therefore not heterogeneity but absence of prespecified measurement; the issue is structural at the design level rather than at the synthesis level (Table 3).

Future investigations employing prospective safety monitoring, standardized adverse event reporting, and repeated‐measures assessment of sensory trick effects will be necessary to characterize both the safety and short‐term durability of sensory trick–based interventions.

## 4 Discussion

This systematic review represents, to our knowledge, the first comprehensive synthesis of evidence on alleviating maneuvers (sensory tricks) in dystonia and the first attempt to formally evaluate the feasibility of quantitative aggregation across this literature. Alleviating maneuvers emerge as a clinically consistent and mechanistically informative phenomenon—observed across dystonia subtypes, replicated across decades of independent studies, and supported by convergent neurophysiological evidence—although the magnitude, durability, and predictors of their effects remain inconsistently measured, and the available evidence does not yet permit reliable pooled estimation across most outcome domains. The nested quantitative synthesis therefore served not only as a methodological strategy but also as a substantive finding by identifying where quantitative synthesis is currently infeasible and highlighting priorities for future research design.

The qualitative synthesis confirms several consistent observations. Sensory tricks occur across dystonia subtypes, most commonly cervical dystonia, and involve diverse modalities including tactile, proprioceptive, visual, cognitive, and imagined maneuvers (Pandey et al. 2017; Masuhr et al. 2000; Singer and Papapetropoulos 2006; Martino et al. 2010; Pandey and Sharma 2018; Norby et al. 2015; Greene and Bressman 1998; Boyd et al. 2013; Lee et al. 2012). Their acute motor effects are clinically meaningful in a substantial proportion of patients, although confidence in the precise magnitude of these effects is constrained by the absence of standardized on–off paradigms and blinded outcome assessment across the contributing studies (see risk of bias section). Kinematic and electromyographic studies often report improvements exceeding 30%–50% in head deviation during trick execution (Müller et al. 2001; Schramm et al. 2004). In dystonic tremor, sensory tricks selectively reduce tremor amplitude without altering tremor frequency, supporting differentiation from essential tremor (Masuhr et al. 2000). These effects are transient and typically decay within minutes after cessation, and they are strongly phenotype‐dependent, being absent or occasionally deleterious in laryngeal dystonia (Dwenger et al. 2025; Avanzino et al. 2025).

The association between sensory trick presence and shorter disease duration, lower baseline severity, and greater responsiveness to botulinum toxin therapy (Mahajan et al. 2023; Kägi et al. 2013; Filip et al. 2016; Tomić et al. 2015) suggests that sensory modulation capacity may reflect a form of neuroplastic reserve that diminishes with disease progression. Supporting this interpretation, pallidal deep brain stimulation can expand the tactile catchment area of sensory tricks (Bain et al. 2009), indicating that the circuits mediating these effects remain accessible to modulation even in advanced disease.

Although the majority of the retrieved literature concerns idiopathic dystonia—most prominently cervical dystonia and blepharospasm—alleviating maneuvers are not restricted to primary forms. They have been documented in inherited dystonias, including pantothenate kinase–associated neurodegeneration, in which a characteristic “forcible” geste antagoniste has been described (Petrović et al., 2014), and in SGCE‐myoclonus‐dystonia, where task‐linked tactile maneuvers are reported during writing and walking (Correa‐Vela et al., 2023). Sensory‐trick–like benefit has also been observed in dystonia secondary to thalamic lesions (Yamada et al., 2007) and in dystonic features within multiple sclerosis (Van der et al., 2015). Collectively, these observations support a network‐level rather than lesion‐specific mechanism: the phenomenon emerges wherever distributed sensorimotor circuits remain accessible to afferent‐driven modulation, regardless of the proximate aetiology of the dystonia.

In contrast, our search did not identify eligible primary studies systematically evaluating alleviating maneuvers in functional or tardive dystonia. This is itself informative. For functional dystonia, the historical use of sensory trick presence as a diagnostic discriminator against psychogenic movement disorders may have discouraged systematic measurement, although contemporary phenomenological work suggests this distinction is not absolute. For tardive dystonia, the relative scarcity of phenomenologically detailed studies—particularly those evaluating volitional sensorimotor strategies—likely reflects the historical emphasis on pharmacological etiology over sensorimotor characterization. Both subgroups represent priority targets for future characterization.

The retrieved literature contains limited but informative evidence on the interaction between dystonia‐directed therapies and alleviating maneuver characteristics. The most consistent signal is bidirectional with botulinum toxin: the presence of an alleviating maneuver is associated with greater subsequent responsiveness to botulinum toxin therapy and lower cumulative dose requirements across focal dystonias (Mahajan et al., 2023; Filip et al., 2016; Tomić et al., 2015), and, conversely, chemodenervation does not appear to abolish the phenomenon itself, suggesting that the underlying sensorimotor mechanism operates upstream of peripheral muscle activity. No included study systematically examined whether oral pharmacotherapy (anticholinergics, benzodiazepines, dopaminergic, or antidopaminergic agents) modifies alleviating maneuver prevalence, magnitude, or durability; this represents an explicit evidence gap given the long‐standing use of these agents in dystonia management. Circuit‐level interventions provide complementary mechanistic insight: pallidal deep brain stimulation can expand the tactile catchment area of an existing alleviating maneuver (Bain et al., 2009), indicating that the circuits mediating these effects remain plastic and accessible to modulation even in advanced disease. The combined picture is one in which alleviating maneuvers index a state of preserved sensorimotor flexibility that can be augmented by—but is not abolished by—current pharmacological and surgical treatments. Whether disease‐modifying or symptomatic therapies preserve, enhance, or accelerate the loss of this flexibility over time is, to our knowledge, untested.

Mechanistically, available evidence supports an afferent‐driven modulation of abnormal sensorimotor integration rather than simple distraction or peripheral muscle inhibition (Avanzino et al. 2025; Cho et al. 2022; Cai et al. 2024; Idrissi et al. 2025; Van der et al. 2015; Correa‐Vela et al., 2023). The implicated neural substrate—including the supplementary motor area, parietal cortex, cerebellum, basal ganglia, and thalamo‐cortical loops—aligns with contemporary network models of dystonia pathophysiology. The persistence of abnormal brainstem and vestibular reflexes during clinical improvement (Mazzini and Schieppati 1994) further indicates that sensory tricks override rather than normalize the underlying pathophysiology. Neurophysiological findings further support cortical involvement: sensory tricks modulate intracortical facilitation (Amadio et al. 2014) and enhance motor preparatory activity reflected in contingent negative variation (Shin et al. 2021), indicating effects on motor planning or prediction rather than simple reflex modulation. The requirement for specific sensorimotor sequences (Filip et al. 2016) also supports higher‐order sensorimotor integration mechanisms.

The mechanistic picture summarized above has direct translational implications for the design of alleviating‐manoeuvre–inspired interventions. Three features of the underlying biology should constrain how such interventions are conceived and evaluated. First, because alleviating maneuvers appear to override rather than normalize the underlying network dysfunction (Mazzini and Schieppati, 1994), device‐based analogues that deliver continuous or repeated afferent input—vibrotactile stimulation (Zhu et al., 2021; Avanzino et al., 2025; Xu et al., 2024; Konczak et al., 2024), mechanical pressure devices (Fantato et al., 2019), and sensory‐trick splints (Yoshida, 2018)—are biologically congruent with the phenomenon they emulate and should not be expected to produce durable carry‐over after stimulation ceases. Second, because the effective neural substrate involves higher‐order sensorimotor integration (SMA, parietal cortex, and cerebellar–thalamo‐cortical loops) (Cho et al., 2022; Cai et al., 2024; Shin et al., 2021), stimulation parameters that engage these networks (e.g., task‐coupled, modality‐matched, and anatomically targeted afferent input) are more likely to reproduce the clinical effect than non‐specific cutaneous stimulation. The selective failure of vibrotactile and auditory manipulations in laryngeal dystonia (Dwenger et al., 2025) illustrates that modality phenotype matching is not optional. Third, because circuit‐level interventions such as pallidal deep brain stimulation can expand the tactile catchment area of an alleviating maneuver (Bain et al., 2009), there is an underexplored opportunity to evaluate device‐based and surgical approaches as complementary rather than competing—for example, using a wearable afferent neuromodulator to test whether a maneuver's effective area or duration can be extended pre‐ or post‐DBS. The single‐blinded parallel‐group randomized trial in the device‐based literature (Konczak et al., 2024) provides a methodological template for the next generation of studies in this space, which should incorporate sham‐controlled designs, instrumented outcomes, and prespecified retention measurements to move the field beyond the proof‐of‐concept stage.

A key finding of this review is that none of the prespecified domains supported formal quantitative pooling. This absence of pooled estimates is itself informative, reflecting structural limitations in study design and reporting rather than an absence of measurable effects. The systematic documentation of domain‐specific barriers—including insufficient numerator–denominator reporting, lack of standardized on–off paradigms, heterogeneous outcome definitions, and limited repeated‐measures designs (Tables 2 and 3)—provides an empirical basis for targeted methodological recommendations.

**TABLE 2:** Evidence gaps and emergent themes identified through qualitative and nested analyses.

Domain/theme	Qualitative findings (what is observed)	Gap Identified (why quantification failed)	Implications for longitudinal Characterization
**Prevalence of sensory tricks**	Sensory tricks are commonly reported across dystonia phenotypes, especially cervical dystonia; prevalence varies widely by phenotype and ascertainment method (spontaneous vs. elicited).	Inconsistent reporting of numerators and denominators; heterogeneous phenotype definitions; dependence on elicitation strategy and assessment context.	Prevalence should be treated as context‐dependent; repeated, standardized assessment over time may distinguish stable traits from state‐dependent reporting.
**Acute motor improvement**	Immediate symptom attenuation during sensory trick execution is consistently described across experimental and clinical studies.	Lack of standardized, same‐session on/off comparisons; heterogeneous outcome measures and responder definitions limit extraction of comparable effect estimates.	Longitudinal designs with harmonized motor metrics could capture within‐subject change and temporal variability in response.
**Tactile sensory tricks (cutaneous afferent modulation)**	Once present, tactile tricks are often reproducible across repeated use and may persist during task execution or continuous stimulation.	Durability assessed inconsistently; multiple dimensions (repeatability, task persistence, and temporal retention) are rarely measured together using comparable metrics.	Longitudinal tracking could disentangle reproducibility, habituation, and true durability as distinct constructs.
**Proprioceptive and postural sensory tricks**	Qualitatively described across phenotypes and often reported as effective in specific contexts or tasks.	Predictor analyses generally examine only the binary presence or absence of sensory tricks rather than objectively measured response magnitude, durability, or task‐specific efficacy; instrumented outcomes are rarely used.	Registries and longitudinal cohorts could link proprioceptive strategies to measurable motor outcomes and evolving response profiles.
**Predictors and modifiers**	Associations reported with disease duration, baseline severity, sensory discrimination, and treatment responsiveness.	Existing analyses evaluate predictors primarily in relation to the presence of sensory tricks rather than quantified motor improvement, durability of benefit, or longitudinal response trajectories.	Repeated assessment would allow predictors to be evaluated against response trajectories rather than cross‐sectional presence alone.
**Device‐based sensory trick analogues**	Early‐phase interventional studies demonstrate short‐term benefit in subsets of patients; interventions are generally well tolerated.	Small sample sizes, heterogeneous devices and stimulation paradigms, short follow‐up periods, and inconsistent outcome definitions limit comparative analysis.	Longitudinal platforms could support comparative effectiveness and durability assessment across device‐based interventions.
**Short‐term retention of effect**	Sensory trick benefits are typically immediate and transient, with improvement occurring during execution and decaying shortly after cessation.	Few studies systematically quantify retention of benefit using standardized time‐based outcome measures following trick cessation or repeated use.	Structured longitudinal assessment could differentiate immediate effects, short‐term retention, and longer‐term adaptation.
**Safety of sensory trick–based interventions**	Clinical reports and interventional studies generally describe sensory tricks and device‐based analogues as well tolerated.	Safety outcomes and adverse events have rarely been prospectively monitored or systematically reported; safety observations are usually incidental to efficacy analyses.	Systematic longitudinal capture would allow differentiation between anecdotal tolerability and well‐characterized safety profiles.
**Mechanistic interpretation**	Convergent evidence supports afferent‐driven modulation within distributed sensorimotor networks involving cortical–subcortical circuits.	Mechanistic studies are often decoupled from clinical durability, functional outcomes, or longitudinal response patterns.	Longitudinal coupling of mechanistic markers with clinical response may clarify adaptive versus compensatory processes.

**TABLE 3:** Summary of extractable quantitative findings by domain.

Domain	Contributing studies (*n*)	Phenotypes represented	Extractable quantitative signal	Heterogeneity barrier preventing pooling	Confidence in directional finding
Prevalence of alleviating maneuvers	16 (with numerator–denominator data in a subset) (Dagostino et al. 2019, Pandey et al. 2017, Norris et al. 2016, Zhou et al. 2016, Matteo et al. 2021, Kilduff et al. 2016, Ehrlich and Frucht 2016, Zhou et al. 2016, Svetel et al. 2013, Idrissi et al. 2025, Van der et al. 2015)	Cervical, blepharospasm, upper‐limb, mixed	Range 13% (spontaneous self‐report, ULD) (Dagostino et al. 2019) to 90% (structured elicitation, BSP) (Pandey et al. 2017); largest registry 68.7% (CD, *n* = 1477) (Norris et al. 2016)	Methodological (ascertainment: spontaneous vs. elicited vs. registry‐coded) and outcome‐level (operational definition of “effective” varies)	Moderate (range, not point estimate)
Acute motor improvement during execution	6 (Pandey et al. 2017, Cutsforth‐Gregory et al. 2016, Fantato et al. 2019, Lorenzano et al. 2019, Correa‐Vela et al. 2023, Velucci et al. 2025) (none meeting pooling criteria)	Predominantly cervical	Improvements > 30%–50% in head deviation reported across kinematic/EMG studies (Müller et al. 2001; Schramm et al. 2004)	**Methodological** (lack of standardized on–off paradigms) and **outcome‐level** (heterogeneous instrumented vs. clinician‐rated outcomes)	Moderate (directional only)
Tactile cutaneous afferent modulation	5 with extractable data (Dagostino et al. 2019, Pandey et al. 2017, Fantato et al. 2019, Correa‐Vela et al. 2023, Erbguth and Lange 2021)	Blepharospasm, cervical, upper‐limb, SGCE‐MD, craniofacial	72.2% (BSP) and 85.8% (CD) effective on every use (Pandey et al. 2017); 38% handwriting improvement with standardized tactile maneuver in ULD (Dagostino et al. 2019); > 50% benefit (18% substantial) with Pressop in BSP, up to 5‐year follow‐up (Fantato et al. 2019)	**Outcome‐level** (sub‐constructs of reproducibility, task‐bound persistence, and long‐term durability are rarely measured together)	Moderate to high (directional)
Proprioceptive/postural maneuvers and predictors	0 meeting eligibility for response‐magnitude synthesis	—	Predictors reported only against binary presence/absence	**Outcome‐level** (binary rather than quantified response)	Low
Device‐based analogues	11 (Amadio et al. 2014; Zhu et al. 2021; Avanzino et al. 2025, Fantato et al. 2019, Lorenzano et al. 2019, Frucht et al. 1999, Yoshida 2018, Nishida et al. 2023, Xu et al. 2024, Konczak et al. 2024, Kasiri et al. 2025) (*n* ≈ 260–280 participants)	Laryngeal, cervical, oromandibular, task‐specific limb, mixed	7/11 with short‐term benefit; responder proportions of 50%–70% were reported (Fantato et al. 2019, Yoshida 2018, Konczak et al. 2024); benefit is transient (minutes to hours, rarely > 24 h)	Methodological (single‐blinded RCT (Konczak et al. 2024); remainder single‐group or unblinded crossover) and clinical (heterogeneous phenotypes and stimulation targets even within vibrotactile subgroup)	Moderate (directional, upper‐bound)
Short‐term retention of effect and safety	0 with prespecified measurement	—	None	**Design‐level absence** of prespecified retention or safety measurement	Insufficient evidence

Abbreviations: BSP, blepharospasm; CD, cervical dystonia; SGCE‐MD, SGCE‐myoclonus‐dystonia; ULD, upper‐limb dystonia.

This pattern reflects a literature that has historically treated sensory tricks as secondary clinical observations rather than primary research outcomes. The prevalence domain illustrates this clearly. Although 16 studies examined sensory trick frequency, only a subset reported extractable numerator–denominator data. Estimates ranged from 13% in spontaneous self‐report in upper limb dystonia 12%–90% in systematically tested blepharospasm (Pandey et al. 2017), while the largest cohort—the Dystonia Coalition registry (*n* = 1477)—reported a prevalence of 68.7% in cervical dystonia (Norris et al. 2016). These differences largely reflect methodological variation rather than true biological inconsistency.

The device‐based sensory trick analoge domain produced the most encouraging translational signal. Across 11 interventional studies enrolling approximately 260–280 participants, seven reported clinically or statistically meaningful short‐term improvements, with responder proportions of 50%–70% where reported (Zhu et al. 2021; Fantato et al. 2019; Lorenzano et al. 2019; Frucht et al. 1999; Yoshida 2018; Nishida et al. 2023; Xu et al. 2024; Konczak et al. 2024). Serious adverse events were not reported, although the absence of prospective safety monitoring limits certainty. With the exception of the single blinded parallel‐group RCT in laryngeal dystonia (Konczak et al., 2024), the predominance of the remaining studies were early‐phase, single‐group, or unblinded crossover designs or uncontrolled designs—without blinded parallel‐group randomized trials—and the reported responder proportions should therefore be regarded as upper‐bound estimates that have not been corrected for placebo or expectation effects (see Risk of Bias). This highlights the need for more rigorous evaluation. An important conceptual distinction is between sensory tricks that override versus those that normalize underlying pathophysiology. The persistence of abnormal vestibular and brainstem reflexes during clinically effective sensory tricks (Mazzini and Schieppati 1994) supports the former interpretation. Consequently, device‐based interventions replicating sensory trick effects may require continuous or repeated afferent input, whereas circuit‐level interventions such as deep brain stimulation may enhance these mechanisms more durably.

Several methodological priorities emerge. Prevalence studies should adopt standardized definitions distinguishing spontaneous self‐report from systematically elicited maneuvers and should report numerator–denominator data stratified by dystonia subtype. Studies examining acute motor effects should employ controlled on–off sensory trick paradigms with objective outcome measures such as kinematics, electromyography, or validated clinical scales. Device‐based interventions should progress toward randomized, sham‐controlled trials with predefined outcomes and structured safety monitoring. Studies examining predictors of sensory trick responsiveness should move beyond binary classification toward quantitative assessment of response magnitude and durability. Future studies should also explicitly characterize alleviating maneuvers in functional and tardive dystonia and prospectively examine the interaction between oral pharmacotherapy and alleviating maneuver characteristics—both of which are currently substantive evidence gaps.

From a clinical standpoint, these findings suggest that alleviating maneuvers (sensory tricks) should be systematically assessed rather than passively awaited, given that prevalence estimates increase markedly—from as low as 13% to over 90%—when structured elicitation is employed rather than spontaneous self‐report. The consistent association between sensory trick/alleviating maneuver presence and botulinum toxin responsiveness further supports their value in prognostic assessment and treatment planning.

Several limitations should be acknowledged. The search strategy was restricted to PubMed, Scopus, and the Cochrane Library; omission of Embase may have limited capture of some European or device‐related literature. The inclusion of Scopus, which indexes a substantial proportion of the same biomedical journal content, partially mitigates this limitation. Only English‐language publications were included. The predominance of observational and exploratory designs, together with small sample sizes in many mechanistic studies, limits the strength of inference. Although the nested meta‐analytic framework was designed to accommodate heterogeneity, no domain ultimately met criteria for pooled analysis. Risk of bias was assessed using design‐appropriate tools, but the diversity of study designs required multiple appraisal instruments, limiting comparability across studies. Publication bias was not formally tested because no domain met the ≥ 10‐study threshold for funnel‐plot–based assessment; small‐study and reporting biases therefore cannot be excluded.

## 5 Conclusions

Sensory tricks—increasingly termed alleviating maneuvers—are a clinically important and mechanistically informative feature of dystonia that have been widely described but inconsistently measured. This systematic review confirms their relevance across dystonia subtypes, their association with favorable prognostic indicators, and their basis in distributed sensorimotor network dysfunction. The nested quantitative synthesis shows that formal meta‐analysis is currently not feasible for most domains because of heterogeneity in study design, outcome definitions, and reporting practices. Early phase evidence supports the translational potential of device‐based alleviating maneuver analogs, although rigorous sham‐controlled evaluation is required before responder estimates can be regarded as confirmed. Together, these findings highlight both the therapeutic promise of alleviating‐maneuver mechanisms and the methodological priorities needed to enable future cumulative quantitative research.

## Author Contributions

**Anish Mehta**: conceptualization, methodology, data curation, writing – original draft, formal analysis, writing – review and editing. **Thyagarajan Shivashanmugam**: methodology, data curation, formal analysis, writing – original draft. **Michiko K. Bruno**: writing – original draft, writing – review and editing. **Roongroj Bhidayasiri**: writing – review and editing. **Sanjay Pandey**: writing – review and editing. **Kailash P. Bhatia**: writing – review and editing. **Pramod Kumar Pal**: writing – review and editing.

## Funding

The authors have nothing to report.

## Ethics Statement

This study is a systematic review of previously published data and did not involve new human participants. Therefore, institutional review board or ethics committee approval was not required.

Informed patient consent was not necessary for this work, as the study involved analysis of previously published data and did not include identifiable patient information.

We confirm that we have read the Journal's position on issues involved in ethical publication and affirm that this work is consistent with those guidelines.

## Conflicts of Interest

The authors declare no conflicts of interest.

## Supporting information




**Supplementary Table S1**: Complete database search strategies and analytic query framework used in the systematic review and nested meta‐analysis.
**Supplementary Table S2**: Characteristics of Included Studies
**Supplementary Table S3**: Risk of bias assessment for the included studies

## Data Availability

All data generated or analyzed during this study are included in this published article and its Supporting Information files. Additional data are available from the corresponding author upon reasonable request.
