# Supplementary material for: Sensory Tricks in Dystonia: A Systematic Review and Nested Quantitative Synthesis
Source: Brain Behav. 2026 Jul 14;16(7):e71575. doi: 10.1002/brb3.71575 (PMC13367128; doi:10.1002/brb3.71575)
Supplement: Supplementary file 1 — Supplementary Table S1: Complete database search strategies and analytic query framework used in the systematic review and nested meta‐analysis. Supplementary Table S2: Characteristics of Included Studies Supplementary Table S3: Risk of bias assessment for the included studies [file BRB3-16-e71575-s001.docx]

# Supplemental Tables

| **Supplementary Table S1. Complete database search strategies and analytic query framework used in the systematic review and nested meta-analysis.** | | |
| --- | --- | --- |
| **Database/ Query** | **Objective** | **Search Strategy / Boolean Syntax** |
| PubMed – Core systematic search | Identify studies evaluating sensory tricks in dystonia | ("Dystonia"[Mesh] OR dystonia[tiab] OR cervical dystonia[tiab] OR blepharospasm[tiab] OR oromandibular dystonia[tiab] OR laryngeal dystonia[tiab] OR focal hand dystonia[tiab] OR segmental dystonia[tiab] OR generalized dystonia[tiab]) AND ("Sensory trick"[tiab] OR "sensory tricks"[tiab] OR "geste antagoniste"[tiab] OR "antagonistic gesture"[tiab] OR "sensory modulation"[tiab] OR "afferent stimulation"[tiab] OR "cutaneous stimulation"[tiab] OR vibrotactile[tiab] OR proprioceptive[tiab] OR tactile[tiab]) |
| PubMed – Prevalence of sensory tricks | Estimate prevalence of geste antagoniste in dystonia | (dystonia[MeSH Terms] OR dystonia*[tiab] OR "cervical dystonia"[tiab] OR torticollis[tiab] OR blepharospasm[tiab] OR "writer* cramp"[tiab] OR "musician* dystonia"[tiab] OR "laryngeal dystonia"[tiab] OR "spasmodic dysphonia"[tiab] OR "oromandibular dystonia"[tiab]) AND ("sensory trick*"[tiab] OR "geste antagoniste"[tiab] OR "antagonistic geste*"[tiab] OR "alleviating maneuver*"[tiab] OR "alleviating manoeuvre*"[tiab]) AND (prevalen*[tiab] OR frequen*[tiab] OR proportion*[tiab] OR occurrence[tiab] OR rate[tiab]) |
| PubMed – Acute motor improvement | Evaluate immediate motor improvement following sensory trick | (dystonia[MeSH Terms] OR dystonia*[tiab] OR "cervical dystonia"[tiab] OR torticollis[tiab] OR blepharospasm[tiab] OR "writer* cramp"[tiab] OR "musician* dystonia"[tiab] OR "oromandibular dystonia"[tiab]) AND ("sensory trick*"[tiab] OR "geste antagoniste"[tiab] OR "antagonistic geste*"[tiab]) AND (acute[tiab] OR immediate[tiab] OR response[tiab] OR responder*[tiab] OR "motor improvement"[tiab] OR posture[tiab] OR EMG[tiab] OR electromyograph*[tiab]) |

| PubMed – Tactile sensory tricks | Identify tactile sensory trick mechanisms | (dystonia[MeSH Terms] OR dystonia*[tiab] OR "cervical dystonia"[tiab] OR torticollis[tiab] OR blepharospasm[tiab] OR "writer* cramp"[tiab] OR "musician* dystonia"[tiab] OR "oromandibular dystonia"[tiab]) AND ("sensory trick*"[tiab] OR "geste antagoniste"[tiab] OR "antagonistic geste*"[tiab]) AND (touch[tiab] OR touching[tiab] OR finger*[tiab] OR hand*[tiab] OR chin[tiab] OR face[tiab] OR cheek[tiab] OR jaw[tiab] OR neck[tiab]) |
| --- | --- | --- |
| PubMed – Proprioceptive/postural tricks | Classification of proprioceptive sensory tricks | No standalone database search |
| PubMed – Device-based analogues | Identify wearable or external sensory modulation devices | (dystonia[MeSH Terms] OR dystonia*[tiab] OR "cervical dystonia"[tiab]) AND ("sensory trick*"[tiab] OR "geste antagoniste"[tiab]) AND (device*[tiab] OR brace*[tiab] OR wearable*[tiab] OR apparatus[tiab] OR vibrotactile[tiab]) |
| PubMed – Short-term retention | Evaluate persistence of sensory trick effects | No standalone database search |
| Cochrane Library | Identify controlled trials and interventional evidence | (dystonia OR "cervical dystonia" OR torticollis OR blepharospasm OR "oromandibular dystonia" OR "laryngeal dystonia" OR "spasmodic dysphonia" OR "writer's cramp" OR "musician's dystonia") AND ("sensory trick" OR "sensory tricks" OR "geste antagoniste" OR "antagonistic geste" OR "afferent stimulation" OR "cutaneous stimulation" OR vibrotactile OR proprioceptive OR tactile) |
| Scopus | Broad indexing search to capture additional records | TITLE-ABS-KEY(dystoni* OR blepharospasm OR torticollis OR "cervical dystonia" OR "focal hand dystonia" OR "writers cramp" OR "writer's cramp" OR "musicians dystonia" OR "musician's dystonia" OR "spasmodic dysphonia" OR "laryngeal dystonia" OR "oromandibular dystonia" OR "cranial dystonia" OR "segmental dystonia" OR "generalized dystonia" OR "generalised dystonia") AND TITLE-ABS-KEY("sensory trick" OR "sensory tricks" OR "geste antagoniste" OR geste OR "alleviating maneuver" OR "alleviating maneuvers" OR "alleviating manoeuvre" OR "alleviating manoeuvres" OR "sensory modulation" OR "sensory stimulation" OR "afferent stimulation" OR "tactile stimulation" OR vibrotactile OR propriocept* OR "cutaneous stimulation") |

| **Supplementary Table 2: Characteristics of Included Studies** | | | | | | |
| --- | --- | --- | --- | --- | --- | --- |
| **Author (Year)** | **Study Design** | **Dystonia Type** | **Sample Size** | **Sensory Trick Modality** | **Outcome Measures** | **Synthesis Category** |
| Amadio et al *et al (*2014)^7^ | Experimental | Cervical dystonia | 8 | Tactile | TMS MEP | Both |
| Avanzino *et al (*2025)^32^ | Interventional | Cervical dystonia | 44 | Vibrotactile | Pain score | Both |
| Benadof *et al (*2019)^33^ | Cross-sectional | Cervical dystonia | 188 | Mixed | TWSTRS CDIP-58 | Qualitative |
| Boyd *et al (*2013)^26^ | Case report | Cervical dystonia | 1 | Visual | EMG video | Qualitative |
| Cai *et al (*2024)^42^ | EEG | Cervical dystonia | 15 | Tactile | Connectivity | Qualitative |
| Cho *et al (*2022)^41^ | fMRI | Cervical dystonia | 23 | Tactile | Connectivity | Qualitative |
| Cisneros *et al (*2020)^45^ | Clinimetric | Cervical dystonia | 185 | Standardized | TWSTRS-2 | Qualitative |
| Correa-Vela *et al (*2023)^56^ | Cross-sectional | Myoclonus dystonia | 49 | Mixed | Clinical scales | Quantitative |
| Cutsforth *et al (*2016)^53^ | Case series | Lower limb | 20 | Mixed | Clinical | Quantitative |
| Dagostino *et al (*2019)^12^ | Case-control | ULD | 37 | Tactile | Handwriting | Quantitative |
| Dwenger *et al (*2025)^31^ | Experimental | Laryngeal | 15 | Multimodal | Voice | Qualitative |
| Ehrlich *et al (*2016)^48^ | Case series | Truncal | 7 | Mixed | Clinical | Quantitative |
| Erbguth *et al (*2021)^58^ | Cohort | Mixed | 101 | Tactile | Symptom change | Quantitative |
| Fantato *et al (*2019)^54^ | Interventional | Blepharospasm | 56 | Device | CDQ-24 | Quantitative |
| Filip *et al (*2016)^35^ | Cross-sectional | Cervical | 197 | Tactile | Questionnaire | Qualitative |
| Frucht *et al (*1999)^59^ | Case series | OMD | 19 | Tactile | Clinical | Quantitative |
| Frucht *et al (*2014)^19^ | Case report | Cervical | 1 | NR | Clinical | Qualitative |
| Greene *et al (*1998)^25^ | Case series | Focal | 3 | Imagined | Clinical | Qualitative |
| Idrissi *et al (*2025)^51^ | Registry | Mixed | 485 | Mixed | Clinical | Quantitative |
| Kagi *et al (*2013)^34^ | Experimental | Cervical | 32 | Multimodal | Thresholds | Qualitative |
| Kasiri *et al (*2025)^64^ | Interventional | Mixed | 28 | Vibrotactile | Motor | Quantitative |
| Kilduff *et al (*2016)^47^ | Cross-sectional | Blepharospasm | 130 | Mixed | Questionnaire | Quantitative |
| Kilic-Berkmen *et al (*2024)^38^ | Cross-sectional | Musician | 365 | NR | Survey | Qualitative |
| Konczak *et al (*2024)^63^ | RCT | Laryngeal | 32 | Vibrotactile | Voice | Quantitative |
| Lee *et al (*2012)^27^ | Case report | Cervical | 1 | Visual | Clinical | Qualitative |
| Lorenzano *et al (*2019)^55^ | Interventional | Blepharospasm | 9 | Device | Clinical | Quantitative |
| Lourenco *et al (*2007)^40^ | Experimental | FHD | 25 | Afferent | MEP | Qualitative |
| Mahajan *et al (*2023)^8^ | Neuroimaging | Cervical | 15 | Tactile | EEG | Qualitative |
| Martino *et al (*2010)^22^ | Cross-sectional | BSP CD | 91 | Mixed | Questionnaire | Qualitative |
| Masuhr *et al (*2000)^20^ | Experimental | Cervical | 60 | Tactile | Accelerometry | Qualitative |

| **Supplementary Table 2 (Continued): Characteristics of Included Studies** | | | | | | |
| --- | --- | --- | --- | --- | --- | --- |
| **Author (Year)** | **Study Design** | **Dystonia Type** | **Sample Size** | **Sensory Trick Modality** | **Outcome Measures** | **Synthesis Category** |
| Matteo *et al (*2021)^46^ | Cross-sectional | Cervical | 57 | NR | Clinical | Quantitative |
| Mazzini *et al (*1994)^44^ | Experimental | Cervical | NR | Indirect | EMG | Qualitative |
| Muller *et al (*2001)^29^ | Observational | Cervical | 50 | Mixed | EMG | Qualitative |
| Murase *et al (*2000)^39^ | Experimental | FHD | 10 | Indirect | SEP | Qualitative |
| Nishida *et al (*2023)^61^ | Case report | Musician | 1 | Electrical | EMG | Quantitative |
| Norby *et al (*2015)^24^ | Case series | Craniocervical | 15 | NR | Clinical | Qualitative |
| Norris *et al (*2016)^14^ | Registry | Cervical | 1582 | NR | GDRS | Quantitative |
| Pandey *et al (*2017)^13^ | Cross-sectional | BSP CD | 40 | Mixed | BFMDRS | Both |
| Pandey *et al (*2018)^23^ | Case series | Meige | 8 | Tactile | BFMDRS | Qualitative |
| Petrovic *et al (*2014)^66^ | Case report | OMD | 1 | NR | Clinical | Qualitative |
| Schramm *et al (*2004)^30^ | Experimental | Cervical | 26 | Tactile | EMG | Qualitative |
| Shin *et al (*2021)^43^ | Experimental | Cervical | 13 | NR | CNV | Qualitative |
| Singer *et al (*2006)^21^ | Comparative | NR | NR | NR | NR | Qualitative |
| Svetel *et al (*2013)^50^ | Genetic | Mixed | 149 | NR | BDNF | Quantitative |
| Tomic *et al (*2015)^36^ | Cohort | Mixed | 100 | NR | TWSTRS | Qualitative |
| Van der Walt *et al (*2015)^52^ | Cross-sectional | MS | 54 | NR | Scores | Quantitative |
| Velucci *et al (*2025)^57^ | Registry | IAOD | 297 | NR | MoCA | Quantitative |
| Xu *et al (*2024)^62^ | Interventional | Cervical | 44 | Vibrotactile | Pain | Quantitative |
| Yamada *et al (*2007)^28^ | Case report | Cervical | 1 | Tactile | TWSTRS | Qualitative |
| Yoshida *et al (*2018)^60^ | Interventional | OMD | NR | Splint | Clinical | Quantitative |
| Zhou *et al (*2016b)^37^ | Genetic | Cervical | NR | NR | NR | Both |
| Zhou *et al (*2016a)^49^ | Genetic | Cervical | 201 | NR | SNP | Quantitative |
| Zhu *et al (*2021)^9^ | Case series | Cervical | 2 | Vibrotactile | Acceleration | Quantitative |

| **Supplementary Table 3: Risk of bias assessment for the included studies** | | | | | | | |
| --- | --- | --- | --- | --- | --- | --- | --- |
| **Author (Year)** | **Study Type** | **Tool** | **Selection Bias** | **Performance Bias** | **Detection Bias** | **Reporting Bias** | **Overall** |
| Amadio et al *et al (*2014)^7^ | Experimental | Cochrane-adapted | Moderate | Moderate | Moderate | Low | Moderate |
| Avanzino *et al (*2025)^32^ | Observational | NOS | Moderate | - | Moderate | Low | Moderate |
| Benadof *et al (*2019)^33^ | Observational | NOS | Moderate | - | Moderate | Low | Moderate |
| Boyd *et al (*2013)^26^ | Case report/series | Checklist | High | - | Moderate | Low | High |
| Cai *et al (*2024)^42^ | Experimental | Cochrane-adapted | Moderate | Moderate | Moderate | Low | Moderate |
| Cho *et al (*2022)^41^ | Experimental | Cochrane-adapted | Moderate | Moderate | Moderate | Low | Moderate |
| Cisneros *et al (*2020)^45^ | Observational | NOS | Moderate | - | Moderate | Low | Moderate |
| Correa-Vela *et al (*2023)^56^ | Observational | NOS | Moderate | - | Moderate | Low | Moderate |
| Cutsforth *et al (*2016)^53^ | Observational | NOS | Moderate | - | Moderate | Low | Moderate |
| Dagostino *et al (*2019)^12^ | Observational | NOS | Moderate | - | Moderate | Low | Moderate |
| Dwenger *et al (*2025)^31^ | Experimental | Cochrane-adapted | Moderate | Moderate | Moderate | Low | Moderate |
| Ehrlich *et al (*2016)^48^ | Observational | NOS | Moderate | - | Moderate | Low | Moderate |
| Erbguth *et al (*2021)^58^ | Observational | NOS | Moderate | - | Moderate | Low | Moderate |
| Fantato *et al (*2019)^54^ | Observational | NOS | Moderate | - | Moderate | Low | Moderate |
| Filip *et al (*2016)^35^ | Observational | NOS | Moderate | - | Moderate | Low | Moderate |
| Frucht *et al (*1999)^59^ | Observational | NOS | Moderate | - | Moderate | Low | Moderate |
| Frucht *et al (*2014)^19^ | Observational | NOS | Moderate | - | Moderate | Low | Moderate |
| **NOS = Newcastle–Ottawa Scale; Cochrane-adapted = domains of selection, performance, detection, and reporting bias; checklist = modified appraisal for descriptive studies.** | | | | | | | |

| **Supplementary Table 3 (Continued): Risk of bias assessment for the included studies** | | | | | | | |
| --- | --- | --- | --- | --- | --- | --- | --- |
| **Author (Year)** | **Study Type** | **Tool** | **Selection Bias** | **Performance Bias** | **Detection Bias** | **Reporting Bias** | **Overall** |
| Greene *et al (*1998)^25^ | Observational | NOS | Moderate | - | Moderate | Low | Moderate |
| Idrissi *et al (*2025)^51^ | Observational | NOS | Moderate | - | Moderate | Low | Moderate |
| Kagi *et al (*2013)^34^ | Experimental | Cochrane-adapted | Moderate | Moderate | Moderate | Low | Moderate |
| Kasiri *et al (*2025)^64^ | Observational | NOS | Moderate | - | Moderate | Low | Moderate |
| Kilduff *et al (*2016)^47^ | Observational | NOS | Moderate | - | Moderate | Low | Moderate |
| Kilic-Berkmen *et al (*2024)^38^ | Observational | NOS | Moderate | - | Moderate | Low | Moderate |
| Konczak *et al (*2024)^63^ | RCT | Cochrane-adapted | Low | Low | Low | Low | Low |
| Lee *et al (*2012)^27^ | Case report/series | Checklist | High | - | Moderate | Low | High |
| Lorenzano *et al (*2019)^55^ | Observational | NOS | Moderate | - | Moderate | Low | Moderate |
| Lourenco *et al (*2007)^40^ | Experimental | Cochrane-adapted | Moderate | Moderate | Moderate | Low | Moderate |
| Mahajan *et al (*2023)^8^ | Observational | NOS | Moderate | - | Moderate | Low | Moderate |
| Martino *et al (*2010)^22^ | Observational | NOS | Moderate | - | Moderate | Low | Moderate |
| Masuhr *et al (*2000)^20^ | Experimental | Cochrane-adapted | Moderate | Moderate | Moderate | Low | Moderate |
| Matteo *et al (*2021)^46^ | Observational | NOS | Moderate | - | Moderate | Low | Moderate |
| Mazzini *et al (*1994)^44^ | Experimental | Cochrane-adapted | Moderate | Moderate | Moderate | Low | Moderate |
| Muller *et al (*2001)^29^ | Experimental | Cochrane-adapted | Moderate | Moderate | Moderate | Low | Moderate |
| Murase *et al (*2000)^39^ | Experimental | Cochrane-adapted | Moderate | Moderate | Moderate | Low | Moderate |
| **NOS = Newcastle–Ottawa Scale; Cochrane-adapted = domains of selection, performance, detection, and reporting bias; checklist = modified appraisal for descriptive studies.** | | | | | | | |

| **Supplementary Table 3 (Continued): Risk of bias assessment for the included studies** | | | | | | | |
| --- | --- | --- | --- | --- | --- | --- | --- |
| **Author (Year)** | **Study Type** | **Tool** | **Selection Bias** | **Performance Bias** | **Detection Bias** | **Reporting Bias** | **Overall** |
| Nishida *et al (*2023)^61^ | Observational | NOS | Moderate | - | Moderate | Low | Moderate |
| Norby *et al (*2015)^24^ | Observational | NOS | Moderate | - | Moderate | Low | Moderate |
| Norris *et al (*2016)^14^ | Observational | NOS | Moderate | - | Moderate | Low | Moderate |
| Pandey *et al (*2017)^13^ | Observational | NOS | Moderate | - | Moderate | Low | Moderate |
| Pandey *et al (*2018)^23^ | Observational | NOS | Moderate | - | Moderate | Low | Moderate |
| Petrovic *et al (*2014)^66^ | Case report/series | Checklist | High | - | Moderate | Low | High |
| Schramm *et al (*2004)^30^ | Experimental | Cochrane-adapted | Moderate | Moderate | Moderate | Low | Moderate |
| Shin *et al (*2021)^43^ | Experimental | Cochrane-adapted | Moderate | Moderate | Moderate | Low | Moderate |
| Singer *et al (*2006)^21^ | Observational | NOS | Moderate | - | Moderate | Low | Moderate |
| Svetel *et al (*2013)^50^ | Observational | NOS | Moderate | - | Moderate | Low | Moderate |
| Tomic *et al (*2015)^36^ | Observational | NOS | Moderate | - | Moderate | Low | Moderate |
| Van der Walt *et al (*2015)^52^ | Observational | NOS | Moderate | - | Moderate | Low | Moderate |
| Velucci *et al (*2025)^57^ | Observational | NOS | Moderate | - | Moderate | Low | Moderate |
| Xu *et al (*2024)^62^ | Observational | NOS | Moderate | - | Moderate | Low | Moderate |
| Yamada *et al (*2007)^28^ | Case report/series | Checklist | High | - | Moderate | Low | High |
| Yoshida *et al (*2018)^60^ | Observational | NOS | Moderate | - | Moderate | Low | Moderate |
| NOS = Newcastle–Ottawa Scale; Cochrane-adapted = domains of selection, performance, detection, and reporting bias; checklist = modified appraisal for descriptive studies. | | | | | | | |

| **Supplementary Table 3 (Continued): Risk of bias assessment for the included studies** | | | | | | | |
| --- | --- | --- | --- | --- | --- | --- | --- |
| **Author (Year)** | **Study Type** | **Tool** | **Selection Bias** | **Performance Bias** | **Detection Bias** | **Reporting Bias** | **Overall** |
| Zhou *et al (*2016b)^37^ | Observational | NOS | Moderate | - | Moderate | Low | Moderate |
| Zhou *et al (*2016a)^49^ | Observational | NOS | Moderate | - | Moderate | Low | Moderate |
| Zhu *et al (*2021)^9^ | Case report/series | Checklist | High | - | Moderate | Low | High |
| NOS = Newcastle–Ottawa Scale; Cochrane-adapted = domains of selection, performance, detection, and reporting bias; checklist = modified appraisal for descriptive studies. | | | | | | | |
